# Danhong Huayu Koufuye Prevents Diabetic Retinopathy in Streptozotocin-Induced Diabetic Rats via Antioxidation and Anti-Inflammation

**DOI:** 10.1155/2017/3059763

**Published:** 2017-05-30

**Authors:** Wenpei Chen, Xiaolan Yao, Chenghao Zhou, Ziyang Zhang, Gang Gui, Baoqin Lin

**Affiliations:** School of Chinese Materia Medica, Guangzhou University of Chinese Medicine, Guangzhou, China

## Abstract

Danhong Huayu Koufuye (DHK), a traditional Chinese prescription, is used to treat central retinal vein occlusion clinically. We previously reported that DHK prevented diabetic retinopathy (DR) in rats. Moreover, we found that it protected endothelial cells from hyperglycemia-induced apoptosis through antioxidation and anti-inflammation. Here, we investigated whether antioxidative and anti-inflammatory activities of DHK contributed to its therapeutic effect on DR in streptozotocin- (STZ-) induced diabetic rats. DHK significantly blocked the breakdown of the blood-retinal barrier (BRB) and increased the thickness of the inner nuclear layer (INL), as well as suppressed the swelling of the ganglion cell layer (GCL) in diabetic retinas. DHK remarkably increased the activities of superoxide dismutase (SOD) and glutathione peroxidase (GPx) in plasma, and decreased serum level of nitric oxide (NO). Moreover, DHK markedly reduced the serum levels of vascular endothelial growth factor (VEGF) and intercellular adhesion molecule-1 (ICAM-1). Furthermore, DHK significantly downregulated protein expressions of VEGF and inducible NO synthase (iNOS) and mRNA expression of ICAM-1 in retinas. These results suggest that the antioxidative and anti-inflammatory activities of DHK may be important mechanisms involved in the protective effect of DHK on DR in STZ-induced diabetic rats.

## 1. Introduction

Diabetic retinopathy (DR), a slow-progressing microvascular complication of diabetes, is the leading cause of blindness among adults aged 20–74 years in the world [1]. The blood-retinal barrier (BRB) breakdown occurs at the early stage of DR [2]. The consequent retinal vascular hyperpermeability results in macular edema, which aggravates the loss of central vision in diabetic patients. BRB disruption attributes to oxidative stress, inflammation, and damage of tight junction integrity, as well as overexpression of vascular endothelial growth factor (VEGF) and advanced glycation end products [3–5].

Hyperglycemia-mediated oxidative damage is a vital pathological mechanism involved in the process of DR [6]. Long-term and persistent hyperglycemia induces the overproduction of reactive oxygen species (ROS) that triggers oxidative stress [7–9]. Low levels or low activities of antioxidative enzymes contribute to further accumulation of ROS, and thus enhance oxidative stress in DR patients [10]. Suppression of oxidative injury inhibits pericyte loss, acellular capillaries formation, capillary basement membrane thickening, and BRB breakdown [11, 12]. Oxidative stress occurs at a very early stage and eventually worsens over the course of diabetes and its complications [13].

Inflammation plays a pivotal role in retinal abnormality in macular edema, ischemia, and neovascularization [14]. There are two pathways associated with inflammation that contribute to BRB breakdown: inflammatory cytokine-induced death of retinal cells pathway and proinflammatory factor-mediated leukostasis and vascular occlusion pathway [15]. Proinflammatory proteins such as inducible nitric oxide synthase (iNOS), intercellular adhesion molecule-1 (ICAM-1), vascular cell adhesion molecule-1 (VCAM-1), and VEGF are highly expressed in diabetic retinas [16–18]. These molecules prompt leukocytes to adhere to the vascular walls and stimulate the release of inflammatory cytokines and vascular permeability factors, which result in BRB breakdown and consequent vascular hyperpermeability [19].

Danhong Huayu Koufuye (DHK), a traditional Chinese prescription, is manufactured by Guangzhou Baiyun Mountain and Hutchison Whampoa Ltd.. It contains 29% *Salviae Miltiorrhizae* Radix et Rhizoma, 11.5% *Angelicae Sinensis* Radix, 15% *Chuanxiong* Rhizoma, 11.5% *Persicae* Semen, 11.5% *Carthami* Flos, 11.5% *Bupleuri* Radix, and 10% *Aurantii* Fructus. DHK alone has a protective effect on DR in streptozotocin- (STZ-) induced diabetic SD rats [20], and it in combination with metformin has a preventive and therapeutic effect on DR in spontaneous type 2 diabetic ZDF rats [21]. Moreover, our previous laboratory study has shown that the antioxidative and anti-inflammatory activities of DHK contribute to protecting endothelial cells from high glucose–induced injury. Furthermore, it inhibits deep venous thrombosis in rats via anti-inflammatory activities [22].

In the present study, we investigated whether antioxidative and anti-inflammatory activities of DHK were involved in the protective effect of DHK on DR in STZ-induced diabetic rats.

## 2. Materials and Methods

### 2.1. Reagents

DHK was kindly provided by Guangzhou Baiyun Mountain and Hutchison Whampoa Ltd. (Guangdong, China). STZ, Evans blue (EB) dye and formamide were purchased from Sigma-Aldrich (California, USA). Pentobarbital sodium salt and xylazine hydrochloride injection were purchased from Merck (Darmstadt, Germany) and Huamu Animal Health Product Co. Ltd. (Jilin, China), respectively. Superoxide dismutase (SOD) and glutathione peroxidase (GPx) assay kits were provided by Nanjing Jiancheng Bioengineering Institute (Jiangsu, China). Thiobarbituric acid reactive substances (TBARS) assay kit was obtained from Cayman Chemical (Michigan, USA). VEGF, ICAM-1, and nitric oxide (NO) assay kits were obtained from R&D Systems (Minneapolis, USA). TRIzol reagent was obtained from Invitrogen (California, USA). M-MLV Rtase cDNA synthesis kit and SYBR Premix Ex Taq II kit were both purchased from Takara Bio Inc. (Dalian, China). Anti-VEGF and anti-iNOS antibodies were bought from Abcam (Cambridge, UK) and Santa Cruz (Texas, USA), respectively. Hematoxylin, eosin, normal goat serum, normal goat IgG, 3% hydrogen peroxide, EDTA antigen retrieval solution, and rabbit anti-IgG staining kit were provided by ZSGB-BIO OriGene (Beijing, China).

### 2.2. Animals

Male SD rats (250 ± 20 g) were provided by the Laboratory Animal Center of Guangzhou University of Chinese Medicine (Guangdong, China). All animals had free access to food and water. They were maintained under a 12 : 12 h cyclic lighting schedule with 22.0–24.0°C and 50–60% humidity. All experiments were performed in accordance with the Local Committee on Animal Care and Use of Guangzhou University of Chinese Medicine (Approval no. SCXK 2013-0020).

### 2.3. Induction of Diabetes and Drug Administration

Rats were fasted for 13 h and then intraperitoneally injected with 60 mg/kg STZ dissolved in citrate buffer (pH 4.3-4.4). Seven days after injection, rats with fasting blood glucose between 16.7 and 33.3 mmol/L were randomly divided into two groups: diabetic control group and DHK treatment group. Age-matched rats were regarded as normal control. The rats in the normal and diabetic control groups were orally administered with distilled water (3.2 mL/kg), and the rats in the DHK treatment group were orally given with DHK (3.2 mL/kg). All rats were treated once daily, consecutively for 16 weeks.

### 2.4. Analysis of BRB Breakdown

After 16 weeks of DHK treatment, the breakdown of BRB in rats was determined as described previously [23]. Firstly, rats were anesthetized with pentobarbital sodium salt (18 mg/kg, intraperitoneally) and xylazine hydrochloride injection (0.19 mL/kg, intramuscularly), and then intravenously injected with EB solution (45 mg/kg). Two minutes later, 0.1 mL blood sample was collected from the iliac artery to obtain the plasma time-averaged EB concentration at a 15 min interval for 2 h. After the final collection of blood, rats were perfused with 37°C citrate-buffered 1% paraformaldehyde for 2 min. Then, the eyes were removed and the retinas were dissected immediately. The retinas were dried, weighed, and incubated in 300 *μ*L formamide at 70°C for 18 h. Finally, the mixture was centrifuged at the speed of 20,000 rpm for 30 min to obtain 180 *μ*L retinal EB solution. The absorbances of the EB solution were taken at 620 nm and 740 nm using a microplate reader (Multiskan GO, Thermo Scientific). The applied absorbance was the difference between these two values. The concentrations of EB in the dry retinas and plasma were calculated from a standard curve of EB. The breakdown of BRB was calculated as follows:

(1) $$\begin{array}{c} BRB\;breakdown=\frac{EB\;\left(\mu g\right)/retina\;dry\;weight\;\left(g\right)}{time-averaged\;EB\;concentration\;\left(\mu g/mL/h\right)}. \end{array}$$

The final results were expressed as the percentage relative to the normal group.

### 2.5. Measurement of SOD and GPx Activities and Malondialdehyde (MDA) Level in Plasma

After 16 weeks of distilled water or DHK treatment, the rats were anesthetized. Fresh arteriopuncture blood samples were then collected into vacutainer with sodium citrate (3.2% w/v) and centrifuged at a speed of 3500 rpm for 10 min. Plasma samples were obtained and used to determine the activities of SOD and GPx and the level of MDA.

### 2.6. Measurement of Serum Levels of VEGF, ICAM-1, and NO

At the end of the experiment, fresh arteriopuncture blood samples were collected into separation gel tubes and centrifuged at a speed of 3500 rpm for 5 min to obtain serum samples. The levels of VEGF, ICAM-1, and NO in serum were determined with commercial assay kits.

### 2.7. Histological Study of the Retina

After blood samples were taken, the right eyes of rats were removed and fixed with 4% paraformaldehyde in phosphate-buffered saline. Paraffin sections (4 *μ*m) of the bulbus oculi were then stained with hematoxylin and eosin (H&E). Pathological pictures of the retinas were taken by an optical microscope (Carl Zeiss, Oberkochen, Germany).

### 2.8. Determination of the Retinal ICAM-1 mRNA Expression

After blood samples were taken, the left eyes of rats were removed and then the retinas were collected. Total RNA was extracted from the retinal tissues using TRIzol reagent. Total RNA samples were quantified spectrophotometrically at 260 and 280 nm, with the A260/A280 ratio ranging from 1.8 to 2.0.

cDNA was synthesized according to the manufacturer's instructions of M-MLV RTase cDNA synthesis kit. The polymerase chain reaction (PCR) was performed using ABI 7500 real-time PCR device (New York, USA) according to the protocol of SYBR Premix Ex Taq II kit. Cycling conditions were as follows: 95°C, 30 s, and 45 cycles of 95°C, 3 s, 60°C, 34 s. The primers of ICAM-1 were GCCTGGGGTTGGAGACTAAC (forward) and CTGTCTTCCCCAATGTCGC (reverse). The primers of GAPDH were AGACAGCCGCATCTTCTTGT (forward) and TGATGGCAACAATGTCCACT (reverse). Quantification of ICAM-1 mRNA was normalized to GAPDH mRNA, and analysis was performed using 2^−ΔΔCt^ method. The final results were expressed as percentage relative to the normal group.

### 2.9. Determination of the Retinal VEGF and iNOS Protein Expressions

The protein expressions of VEGF and iNOS in the retinas were assessed by immunohistochemical assay. Paraffin sections (4 *μ*m) of the bulbus oculi were blocked with 3% hydrogen peroxide for 10 min, and then underwent heat treatment with EDTA antigen retrieval buffer solution for 15 min. When slides were cooled to the room temperature, the retinal tissues were blocked with normal goat serum for 10 min and then incubated with primary antibodies of VEGF (ab46154, 1 : 200 dilution) or iNOS (sc-649, 1 : 50 dilution) at 4°C overnight. Rabbit anti-IgG staining kit was used in subsequent steps according to the manufacturer's instructions. Normal goat IgG was used as a negative control. Six pathological pictures of each retina were taken by an optical microscope (Carl Zeiss, Oberkochen, Germany). These pictures were then analyzed with the Image-Pro Plus Analysis Software 6.0 (Rockville, MD, USA).

### 2.10. Statistical Analysis

Data were expressed as mean ± standard error of the mean (SEM) and analyzed by Statistical Package for the Social Sciences version 21.0 (SPSS 21.0). One-way analysis of variance (ANOVA) test was performed, and post hoc multiple comparisons were conducted with LSD. *P* < 0.05 was regarded to be significant.

## 3. Results

### 3.1. DHK Blocks BRB Breakdown in STZ-Treated Rats

**Figure 1:**
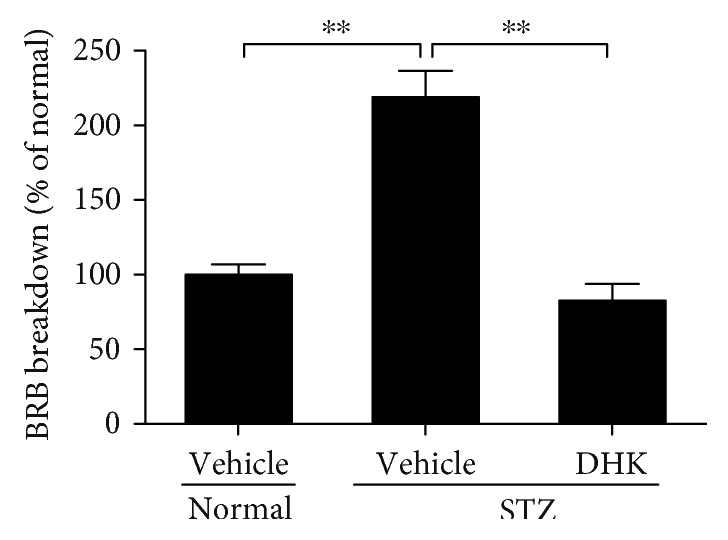
Effect of DHK on the BRB breakdown in rats. Seven days after injection of STZ, rats were orally administered for 16 weeks. At the end of the experiment, the breakdown of BRB in rats was determined using EB dye. Results were expressed as percentage relative to the normal group. Values were expressed as mean ± SEM, *n* = 8–10, ^∗∗^*P* < 0.01.

As shown in Figure 1, BRB permeability was significantly increased by 118.9% in diabetic control rats (*P* < 0.01 versus normal control rats). DHK significantly decreased the breakdown of BRB by 136.3% (*P* < 0.01 versus diabetic control group).

### 3.2. DHK Attenuates Oxidative Stress in STZ-Treated Rats

**Figure 2:**
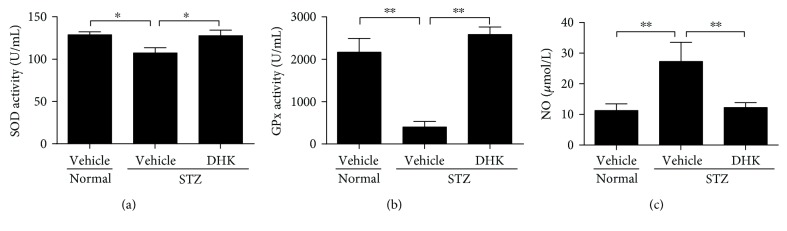
Effects of DHK on plasma activities of SOD (a) and GPx (b) and serum level of NO (c) in rats. Seven days after injection of STZ, rats were orally administered for 16 weeks. At the end of the experiment, rats were anesthetized. Blood samples were collected to obtain plasma or serum. The activities of SOD and GPx and the level of NO were measured with commercial assay kits. Values were expressed as mean ± SEM, *n* = 8–10, ^∗^*P* < 0.05, and ^∗∗^*P* < 0.01.

As compared with normal control rats, the plasma activities of SOD and GPx in diabetic control rats were markedly decreased by 16.8% (*P* < 0.05) and 81.5% (*P* < 0.01), respectively, while serum NO level was significantly increased by about 1.4-fold (*P* < 0.01). DHK significantly increased SOD activity and decreased NO level by 19.1% (*P* < 0.05 versus the diabetic control group) and 55.1% (*P* < 0.01 versus the diabetic control group), respectively (Figures 2(a) and 2(c)). DHK remarkably enhanced GPx activity by about 5.5-fold (*P* < 0.01 versus the diabetic control group; Figure 2(b)). There were no differences among groups in the MDA level (data not shown).

### 3.3. DHK Decreases Serum Levels of VEGF and ICAM-1 in STZ-Treated Rats

**Figure 3:**
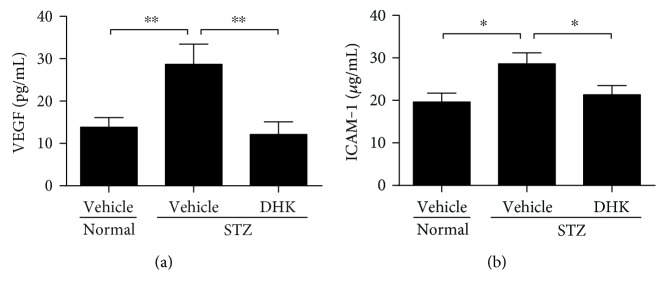
Effects of DHK on serum levels of VEGF (a) and ICAM-1 (b) in rats. Seven days after injection of STZ, rats were orally administered for 16 weeks. At the end of the experiment, rats were anesthetized. Blood samples were collected to obtain serum. Levels of VEGF and ICAM-1 were measured with commercial assay kits. Values were expressed as mean ± SEM, *n* = 8–10, ^∗^*P* < 0.05, and ^∗∗^*P* < 0.01.

As compared with normal control rats, serum levels of VEGF and ICAM-1 in diabetic rats were markedly increased by about 107.3% (*P* < 0.01) and 45.9% (*P* < 0.05), respectively (Figure 3). DHK treatment notably decreased VEGF and ICAM-1 levels by 57.9% (*P* < 0.01 versus the diabetic control group) and 25.5% (*P* < 0.05 versus the diabetic control group), respectively.

### 3.4. DHK Protects the Retinal Structure and Downregulates the Retinal Protein Expressions of VEGF and iNOS in STZ-Treated Rats

**Figure 4:**
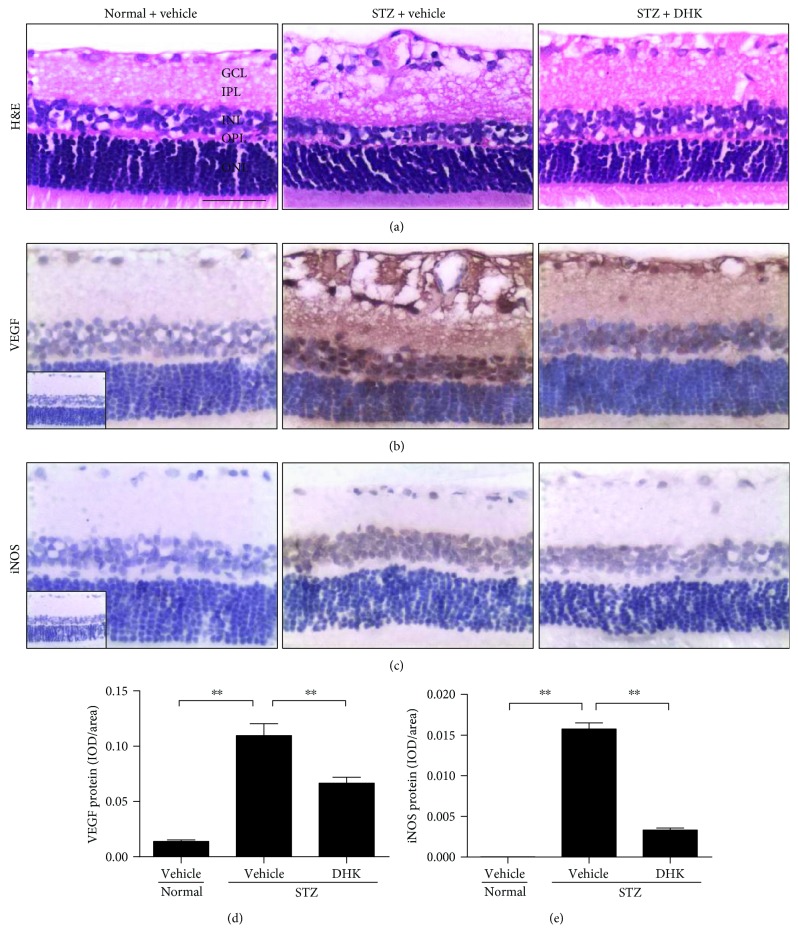
Effects of DHK on morphological changes (a), protein expressions of VEGF (b and d), and iNOS (c and e) in diabetic retinas. Seven days after injection of STZ, rats were orally administered for 16 weeks. At the end of the experiment, rats were sacrificed, and the right eyeballs were removed and then fixed with 4% paraformaldehyde in phosphate-buffered saline. Paraffin sections (4 *μ*m) of the bulbus oculi were then stained with H&E or applied for immunohistochemical studies. GCL: ganglion cell layer; IPL: inner plexiform layer; INL: inner nuclear layer; OPL: outer plexiform layer; ONL: outer nuclear layer; IOD: integral optical density. Scale bar: 50 *μ*m. Values were expressed as mean ± SEM, *n* = 8–10, and ^∗∗^*P* < 0.01.

In rodents, the retina is composed of five basic layers: ganglion cell layer (GCL), inner plexiform layer (IPL), inner nuclear layer (INL), outer plexiform layer (OPL), and outer nuclear layer (ONL) [5]. Thin IPL and INL and swollen GCL were presented in the retinas of diabetic control rats. DHK increased the thickness of INL and improved the swollen GCL (Figure 4(a)).

High VEGF expression was mainly identified in GCL, IPL, and INL in diabetic retinas, whereas weak staining was observed in normal retinas (Figures 4(b) and 4(d)). iNOS was abnormally overexpressed in GCL, IPL, and INL in diabetic retinas (Figures 4(c) and 4(e)). DHK remarkably downregulated these two protein expressions in diabetic retinas (Figures 4(d) and 4(e)).

### 3.5. DHK Downregulates the Retinal ICAM-1 mRNA Expression in STZ-Treated Rats

**Figure 5:**
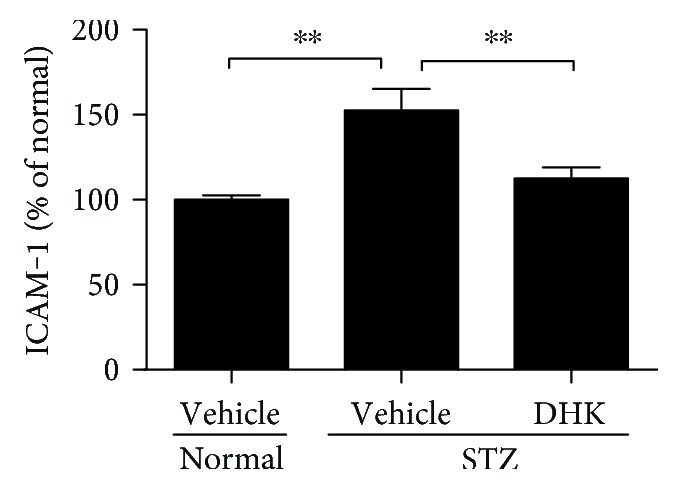
Effect of DHK on mRNA expression of ICAM-1 in the retinas. Seven days after injection of STZ, rats were orally administered for 16 weeks. At the end of the experiment, rats were sacrificed and the left retinas were then collected. Retinal total RNA was extracted from the retina using TRIzol reagent. Retinal mRNA expression of ICAM-1 was evaluated using real-time PCR method. Results were expressed as percentage relative to the normal group. Values were expressed as mean ± SEM, *n* = 8–10, and ^∗∗^*P* < 0.01.

Diabetic control rats presented a marked upregulation of ICAM-1 mRNA (*P* < 0.01 versus the normal control group). DHK significantly decreased mRNA expression of ICAM-1 (*P* < 0.01 versus the diabetic control group; Figure 5).

## 4. Discussion

The BRB consists of vascular endothelial cells and retina pigment epithelium cells. The differentiation of endothelial cells requires interaction with the glial cells and pericytes [5]. Therefore, the injury of these cells results in retinal morphological changes such as edema and/or thinness [5, 24], which contribute to vision loss in DR. In the present study, DHK significantly decreased the breakdown of BRB, increased the thickness of INL, and inhibited the swelling of GCL (Figures 1 and 4(a)), which reveals that the protective effect of DHK on the retinal structure helps to reduce the BRB breakdown.

Oxidative stress is considered as an important factor in the pathology of DR. Persistent hyperglycemia induces ROS formation and triggers oxidative stress. ROS injures the retinal endothelial cells through damaging proteins, lipids, and DNA [25, 26]. ROS-mediated damage of the retinal cells forms a special memory in the microvasculature, so this damage sustains even when glucose has been normalized [27].

In addition to ROS, low-level defense system is another key point responsible for the imbalance of the redox status and oxidative stress in DR. Antioxidant enzymes such as SOD, GPx, and catalase (CAT) are the most important components of the defense system and take active part in ROS homeostasis. SOD is the first antioxidant enzyme against ROS. It coverts O^2−^ into H_2_O_2_, which is further degraded into O_2_ and H_2_O by GPx or CAT [26, 28]. A remarkable decline of SOD, GPx, and CAT was found in DR patients, animals, and high glucose–induced retinal endothelial cells [10, 28, 29]. DHK significantly increased the activities of SOD and GPx to normal level (Figures 2(a) and 2(b)). These results suggest that DHK has a protective effect on BRB via reducing oxidative stress.

Not only oxidative damage but also inflammation is responsible for the process of DR. ICAM-1 is highly expressed in diabetic endothelial cells [30]. It mediates the interaction between leukocytes and capillary endothelial cells and then initiates inflammatory response [31]. CD18, a ligand of ICAM-1, remarkably arises in patients with each stage of DR [32] and contributes to leukostasis. Diabetic ICAM-1- or CD18-deficient mice are protected from leukostasis, endothelial cells injury, pericyte loss, acellular capillaries, and BRB breakdown [33]. Thus, ICAM-1 is an important proflammatory factor in DR.

Intraocular VEGF expression is markedly upregulated in diabetic patients, rats, and mice [18, 34, 35]. VEGF damages BRB not only through breaking endothelial tight junctions, increasing vascular permeability, and leading to retinal edema but also through inducing leukocyte aggregation, touching off inflammatory response, and injuring endothelial cells [3]. VEGF induces high expression of ICAM-1 in the retina and then activates the adhesion of leukocytes [36]. The inflammation in turn stimulates further release of VEGF [19, 36], which eventually leads to the breakdown of BRB. DHK significantly downregulated VEGF and ICAM-1 expressions (Figures 3, 4(d), and 5). These results suggest that DHK protects BRB via anti-inflammation.

NO is generated by neuronal NOS (nNOS), endothelial NOS (eNOS), and iNOS. Increased serum NO level is associated with increased severity of DR since it breaks reactive nitrogen species homeostasis and injures the retinal structure [37, 38]. The function of endothelial cells is damaged, and subsequently, eNOS is downregulated in diabetic patients and animals. So, the increased serum NO may be from iNOS [39]. Retinal iNOS is mainly expressed in IPL, INL, and OPL and is upregulated in diabetic ones [40–42]. Diabetic iNOS knockout mice are protected from leukostasis and BRB breakdown because of low expression of ICAM-1 and high expressions of tight junction proteins [41]. DHK significantly decreased serum NO level and reduced the retinal iNOS protein expression (Figures 2(c) and 4(e)), which indicates that DHK protects the integrity of BRB via regulating nitrogen species homeostasis, reducing oxidative stress, and ameliorating inflammatory response.

## 5. Conclusion

In summary, DHK ameliorates BRB breakdown and protects the retinal structure in STZ-induced diabetic rats. DHK enhances the activities of SOD and GPx and inhibits the production of NO, VEGF, iNOS, and ICAM-1. These results suggest that the antioxidative and anti-inflammatory activities of DHK may be important mechanisms involved in the protective effect of DHK on DR in STZ-induced diabetic rats.
